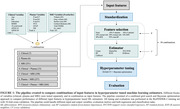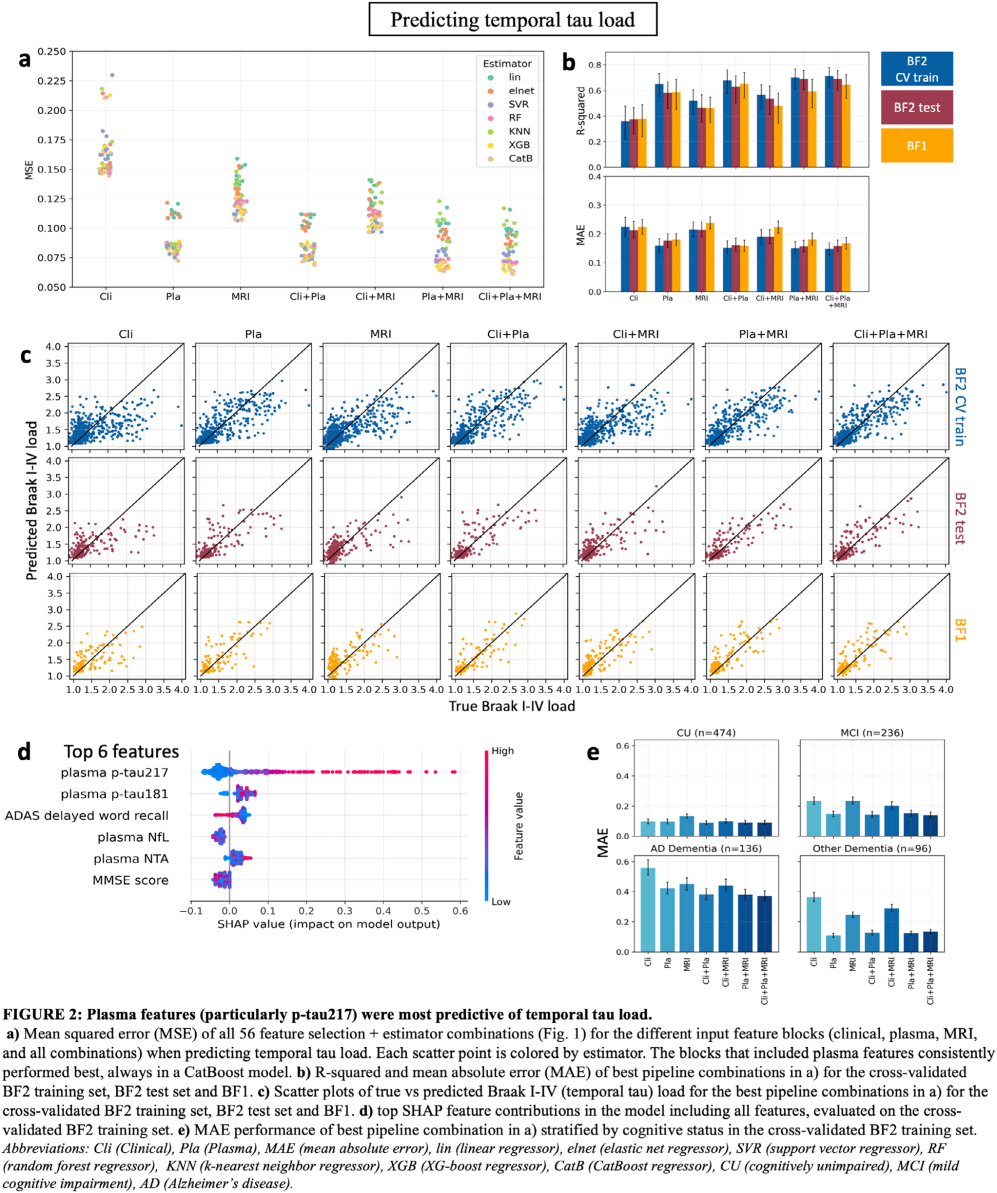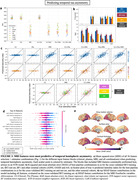# Unlocking Tau PET Accessibility: a Machine Learning‐Based Prediction of Tau Pathology from Plasma, MRI and Clinical Variables

**DOI:** 10.1002/alz.089129

**Published:** 2025-01-09

**Authors:** Linda Karlsson, Jacob W. Vogel, Olof Strandberg, Ida Arvidsson, Kalle Åström, Jakob Seidlitz, Richard A.I. Bethlehem, Erik Stomrud, Rik Ossenkoppele, Nicholas J. Ashton, Kaj Blennow, Sebastian Palmqvist, Ruben Smith, Shorena Janelidze, Alexa Pichet Binette, Niklas Mattsson‐Carlgren, Oskar Hansson

**Affiliations:** ^1^ Clinical Memory Research Unit, Lund University, Lund Sweden; ^2^ Department of Clinical Sciences Malmö, SciLifeLab, Lund University, Lund Sweden; ^3^ Clinical Memory Research Unit, Department of Clinical Sciences Malmö, Faculty of Medicine, Lund University, Lund Sweden; ^4^ Centre for Mathematical Sciences, Lund University, Lund Sweden; ^5^ Penn/CHOP Lifespan Brain Institute, University of Pennsylvania, Philadelphia, PA USA; ^6^ University of Cambridge, Cambridge UK; ^7^ Memory Clinic, Skåne University Hospital, Malmö Sweden; ^8^ Alzheimer Center Amsterdam, Amsterdam UMC, Amsterdam Netherlands; ^9^ Institute of Neuroscience and Physiology, Sahlgrenska Academy at the University of Gothenburg, Gothenburg Sweden; ^10^ Institute of Psychiatry, Psychology and Neuroscience, Maurice Wohl Clinical Neuroscience Institute, King’s College London, London UK; ^11^ Clinical Neurochemistry Laboratory Sahlgrenska University Hospital, Mölndal Sweden; ^12^ Institute of Neuroscience and Physiology, Sahlgrenska Academy at the University of Gothenburg, Göteborg Sweden; ^13^ Skåne University Hospital, Lund Sweden; ^14^ Department of Neurology, Skåne University Hospital, Lund Sweden; ^15^ Clinical Memory Research Unit, Department of Clinical Sciences, Lund University, Lund Sweden

## Abstract

**Background:**

A key characteristic of Alzheimer’s disease (AD) is cerebral aggregation of tau. These aggregates can be quantified and localized with positron emission tomography (PET), which improves the diagnostic and prognostic work‐up of AD. However, tau‐PET is expensive and not available in clinical settings globally. With emerging disease‐modifying therapies for AD, comprehensive and accessible ways to estimate the load and distribution of tau pathology are becoming crucial. Here, we address this problem with machine learning (ML) models trained to predict tau‐PET outcomes from accessible features only (clinical variables, plasma biomarkers and MRI).

**Method:**

The study included 1195 participants from the BioFINDER‐2 (BF2) cohort. BF2 was split into 80% train and 20% test sets. We created a rigorous ML pipeline that, with 10‐fold cross‐validation, evaluated different feature selection steps and estimators in a combined grid‐search and Bayesian optimization set‐up (Figure 1). The three variable blocks (clinical, plasma and MRI) were used as features (together and separately) to predict two tau‐PET characteristics: 1) tau load in the temporal cortex 2) hemispheric asymmetry of temporal tau load in tau positive participants (as an example of clinically relevant spatial information). External validation was performed in the BF2 test set, BioFINDER‐1 (BF1, n=147), ADNI (n=136), OASIS (n=46) and A4 (n=45). Note that ADNI, OASIS and A4 did not include plasma biomarkers.

**Result:**

Feature combinations including plasma (particularly plasma p‐tau217) resulted in the best prediction of tau‐PET load (Figure 2). The best performing model was a combination of plasma and MRI variables in a CatBoost regressor, which achieved SUVR mean absolute error (MAE) = 0.155 and R‐squared = 0.700 on unseen data. For prediction of hemispheric asymmetry, models using MRI features performed best, and regions in the temporal lobe had the greatest contribution to the models (Figure 3). Top performance on unseen data was laterality index MAE = 6.74 and R‐squared = 0.423 in a SVR model including MRI and clinical variables.

**Conclusion:**

Generalizable machine learning models reveal the potential in predicting tau PET from more accessible variables (particularly plasma biomarkers and MRI), which can unlock global accessibility to one of the most informative in vivo diagnostic techniques of AD.